# Does the student-led osteopathy clinical learning environment prepare students for practice?

**DOI:** 10.1186/s12909-022-03658-3

**Published:** 2022-08-05

**Authors:** Conor Abrey, Niraj De Silva, Jake Godwin, Thomas Jacotine, Daniel Raab, Kieran Urquhart, Kelley Mumford, Patrick McLaughlin, Brett Vaughan

**Affiliations:** 1 College of Health & Biomedicine, Victoria University, Melbourne, Australia; 2 Institute for Health and Sport, Victoria University, Melbourne, Australia; 3 Department of Medical Education, University of Melbourne, Melbourne, Australia; 4 Faculty of Health, Southern Cross University, Lismore, Australia

**Keywords:** Student-led clinic, Clinical educator, Osteopathy, Capabilities

## Abstract

**Background:**

For many allied health disciplines, pre-professional clinical education takes place in student-led, on-campus clinic environments. In these environments, pre-professional students undertake patient care under the supervision of qualified health professionals. Literature exploring the benefits of the student-led clinical learning environment is limited and little is known about the role student-led clinics play in preparing pre-professional osteopathy students for professional practice.

**Aim:**

To explore the perceptions of osteopathy clinical educators about the role of the student-led clinic at Victoria University (VU) in preparing pre-professional students for professional practice.

**Methods:**

A qualitative collective case study methodology was utilised to explore clinical educator perceptions. Individual interviews were conducted with clinical educators employed in the university osteopathy clinic. Interview questions were framed around the Capabilities for Osteopathic Practice which set the Australian osteopathy practice standards. Data were assessed by two of the authors using thematic analysis.

**Results:**

Nine clinical educators out of 31 employed at the university clinic (29%) agreed to participate. Qualitative analysis generated three themes: perceptions of the student-led clinic (SLC) as a learning environment; clinical educator perception of their role in the SLC; and, challenges to and of the SLC environment.

**Conclusions:**

Clinical educators perceived that the student-led osteopathy clinical learning environment develops pre-professional learners to meet some, but not all, of the capabilities for professional practice as an osteopath in Australia. The environment may be improved through faculty development, fostering a proactive learning approach, addressing system-based issues, and providing opportunities to interact with other health professions.

## Introduction

Pre-professional clinical education typically takes place in primary or tertiary health care settings. These student clinical placement opportunities differ considerably in format and duration within, and amongst, the health professions. Variations in supervision style, scheduling and timing of placements exist across the spectrum of pre-professional training courses in Australia [1–5]. Variations also exist in the patient base that presents to these students. Where once clinical placements were the domain of the hospital setting, clinical education in allied health is not limited to large city hospitals with affiliated medical schools [6]. A proliferation of, and demand for, allied health courses and services in Australia [7] has necessitated an increase in the diversity of settings for clinical placements to include student-led clinics (SLC) based on university campuses and in community health centers [8–10].

Student-led clinics provide a clinical setting in which students can develop the skills, attitudes, values, knowledge and capabilities for their profession, preparing them for professional practice [9, 11]. The central premise of all SLCs is that the student practitioner, supported by a qualified clinical educator, is the main provider of care for the patient [12, 13]. In Australia, SLCs are commonly housed on a university campus, but this is not the only clinical learning environment with which students engage. The provision of care to under-served or lower socio-economic populations through community health centres or outreach clinics [14], also provide diverse experiences for the pre-professional allied health student [15]. These external opportunities help to address placement shortages that exist in health professions due to an increase in demand for student placements [9, 11, 16, 17].

Student satisfaction is overwhelmingly positive in regards to the learning experience in the SLC setting [15]. Students report that their clinical decision making, history taking, physical examination and patient ownership skills are improved due to the SLC environment and that these skills are unlikely to be acquired or developed in another environment (e.g. classroom) [11, 18]. Further, Fröberg, Leanderson, Fläckman, Hedman-Lagerlöf, Björklund, Nilsson and Stenfors [12] reported that patients perceived student practitioners demonstrated a high quality of care and professionalism, whilst Schutte, Tichelaar, Dekker, van Agtmael, de Vries and Richir [11] reported some patients suggest there is a better quality of care in SLCs compared to regular health services.

Although SLCs assist the development of essential skills in student health professionals, there are also perceived disadvantages. Students report that SLCs do not cover the full range of problems and treatments regarded as important to their professional development. These issues include limited development in professional communication, exposure to a variety of client/patient problems, and a lack of authenticity during a consultation especially when the patients being treated are students themselves or do not have an actual health problem [9, 10, 19].

Australian osteopaths are government-registered health professionals who mostly work in the private practice setting [20]. To be eligible for registration, they are required to complete an accredited university program [21]. In Australian osteopathy programs, students undertaking placements in SLCs are supervised by registered, practicing osteopaths who provide appropriate support and guidance [22, 23]. The attributes, knowledge and skills required for Australian osteopaths to practice safely and ethically are described in ‘The Capabilities of Osteopathic Practice’ (Osteopathy Australia, 2020). This document highlights seven integrated roles (osteopath, professional and ethical practitioner, communicator, critical reflective practitioner and lifelong learner, educator and health promoter, collaborative practitioner, and leader and manager) along with the key capabilities and enabling components for each role. The format of these Capabilities is consistent with the CanMEDs [24] and sets the standards against which university programs are designed and graduates assessed.

It is not clear in the current literature how SLCs specifically contribute to the development of students’ capabilities for practicing as an osteopath. Current knowledge of the effectiveness of osteopathy SLCs mostly focuses on student and patient perceptions [10, 25, 26]. The perceptions of the clinical educator – the registered professional who guides students through their SLC experience – has not previously been canvassed. This study sought the opinion of Australian osteopathic clinical educators concerning the utility of SLCs to prepare pre-professional students for professional practice.

## Methods

### Study approach

This study utilised a collective case study design and convenience sampling. The ‘collective’ in this design refers to the fact that multiple cases are being examined [27]. A case study is an effective study design when the research is targeted towards explaining, describing, or exploring events or phenomena within the everyday context. This form of research allows the study to answer questions of ‘how’, ‘what’, and ‘why’ to gain a deeper understanding of what gaps exist in the chosen topic of the research [28]. The study was approved by the Victoria University Human Research Ethics Committee (HRE20-170).

### Context

Students in the Victoria University (Melbourne, Australia) osteopathy course undertake the majority of their clinical education at an on-campus SLC [22]. They also have the option to provide care to under-serviced populations through community-based clinics. The on-campus SLC is open to the general public and patients present with various musculoskeletal complaints [29], to be treated by students at a reduced cost compared to that of a standard osteopathy service [22]. Osteopathy clinical educators at the institution are registered, practicing osteopaths with at least 3 years clinical experience. The role of the clinical educator is to guide, support and provide ongoing feedback to the students, ensuring that they are consulting in a safe and effective manner. These educators are employed on a casual basis in four-hour blocks in order to provide supervision coverage 5 days per week. A summary of the practice and patient management of Australian osteopathy clinical educators is provided elsewhere [23].

### Participants

Potential participants in the study were known to the authors either through their professional or educational relationships. Participants were provided with both a summary of the study in the information to participant’s sheet and also oriented to the study at the beginning of the interview. This orientation also included a description of the research teams’ interest in the study alongside the interviewer’s interest. The interviewer (BV) had previously been a clinical coordinator in the learning environment that was of interest in the current work, and began their PhD work during this time. However, this role was completed in 2016 and the interviewer did not have a subsequent role in the same environment.

The participants for this study were current osteopathy clinical educators employed by the institution, with recruitment occurring from November 2020 to February 2021. Participants were recruited through an email invitation from one of the researchers (PM). Interested participants were invited to indicate their intent to participate by return email. Each participant was sent an additional email with an informed consent document that they signed before data collection commenced.

### Interviews

Participants undertook a semi-structured interview conducted by one male author, BV. The author (BV) has a PhD in clinical education in addition to health professions and public health qualifications. At the time of the study, the author was a qualified osteopath and lecturer in clinical education at the University of Melbourne. The author who conducted the interviews was experienced with both quantitative and qualitative designs, including leading focus groups and one-on-one interviews.

Each interview was up to 1 h in duration and conducted via the Zoom video conference platform. The interview was audio-recorded and auto-transcribed using Otter (www.otter.ai). Transcribed interviews were allocated a number, then edited to de-identify the participant, other people or organisations, and to ensure accuracy of the transcription.

### Analysis

Initial analysis of the data was undertaken by two of the authors and followed the process described by Braun, Clarke, Hayfield and Terry [30]: familiarisation with the data as the researchers independently read through the transcripts; coding with succinct labels that identified important features relating to the research question to create codes focusing on the individual capabilities; initial themes were generated using the codes and collated data which resulted in; the reviewing of themes and generation of overarching themes; these themes were then named and defined to identify the scope and focus of each theme.

As all authors had access to the interview transcripts, the analytic narrative and data extracts were discussed to produce consensus on common codes and themes. The final phase involved bringing together the analytic narrative and data extracts to be able to relate the results to existing literature.

Trustworthiness of the current work focused on the areas described by Lincoln & Guba [31] including credibility, dependability, confirmability, and transferability. Credibility was established through engagement with the participants for a duration of their choosing in order to provide fulsome responses to the interview questions. Further, the data were analysed by multiple authors with the themes checked and rechecked against the data to support their development. Dependability was established through the participants being invited to respond to questions about broad experiences that were unlikely to change over the period of the study. For confirmability, regular discussions about the analysis were undertaken between the authors and the two senior authors (BV and PMcL) debriefed after each interview. With respect to transferability, interview questions were designed so that they could be applicable to other student-led osteopathy clinical learning environments, and the participants were reflective of those who would participate in this environment as clinical educators.

## Results

Nine of a possible 31 clinical educators (29%) agreed to participate in the study. Data of nine participants was deemed to be an acceptable sample size due to the consistency of the concepts described by the participants indicating that the data had reached saturation [32]. Table 1 provides the gender and number of years of experience as a clinical educator of each participant.

**Table 1:** Overview of participant gender and experience as an osteopathy clinical educator

Participant	Gender and Years as a Clinical Educator
1	Male, four years
2	Female, six years
3	Male, three years
4	Male, ten years
5	Female, three years
6	Male, five years
7	Male, five years
8	Male, greater than ten years
9	Female, five years

Three themes were identified based on the participant responses: perceptions of the SLC as a learning environment; clinical educator perception of their role in the SLC; and, challenges to and of the SLC environment.

### Perceptions of the SLC as a learning environment

The clinical educators consider the SLC to be an effective environment for students to consolidate their treatment skills. The ability of students to create management plans based on clinical reasoning and scientific evidence was a common thread among the clinical educators. This included staying within their scope of practice by developing referral skills.

*“[Students are] good at prescribing rehab [rehabilitation] and exercise plans. [city-based clinic] has access to a gym which students implement for patients’ management plan.” (Interviewee 4).*

*“EBP tasks are great for students as it reinforces best management plans. Good way to expose to clinical guidelines and implement an EBP.” (Interviewee 1).*

Clinical educators suggested the SLC was a suitable environment for students to consolidate key skills described within the Capabilities for Osteopathic Practice, particularly consolidating verbal and non-verbal communication skills. Being able to take thorough clinical notes in a timely fashion is another skill that the SLC helps to develop.

*“Clinic does a great job and promotes having to present to their clinician and communication is a super important skill.” (Interviewee 1).*

*“…we’re talking about professional communication, meaningful communication, where you’re establishing relationships that are, you know, patient-practitioner relationships, not friend relationships.” (Interviewee 8).*

*“So I think of it that immersion into clinic in those, especially those first couple of months, where they’re trying to take histories, and you can see that because they start out, and they’ve got an hour and a half appointment, and their clinical history for a new patient takes them an hour. They start to really develop communication skills, and they can talk to their patients, they can talk to their peers, and they can offer feedback to younger students.” (Interviewee 2).*

The SLC was viewed by the clinical educators as being a safe environment to develop students’ professional and ethical behaviours, whilst also being an appropriate environment to become an effective health promoter. This was further enhanced in community clinics where the patient demographic/population is more vulnerable.

*“Student clinic is a good place to develop ethical decision making. Really good with the community clinics. There is a lot more opportunity to develop in community clinics as opposed to the city clinic.” (Interviewee 5).*

*“The clinic is a good spot to practice being an educator and health promoter. Students should get better at promoting the profession and the student clinic by learning how you might get patients and clients to come in and see you.” (Interviewee 7).*

### Clinical educator perception of their role in the SLC

Clinical educators described how they play a role in fostering a learning environment that assists in the preparation of students for clinical practice. Reflective practice was described by the participants as one of the key areas where they can play a role in developing and encouraging learning.

*“Perfect environment for it [reflective practice]. But again, it needs to be kind of implemented and encouraged in a structured way.” (Interviewee 5).*

*“I guess that might have to come from the clinician side as far as asking them. But there might be something where they will be forced to reflect, like you said on each patient and, and know what they did well, and what they could improve on.” (Interviewee 4).*

Evidence-based practice (EBP) tasks were identified as a potential strategy to engage students with reflective practice in the aforementioned “structured way”. Students are required to complete an EBP task as part of their patient management. The participants appeared to focus on reflection as it relates to the treatment and management aspects of the consultations undertaken by students, rather than the consultation as a whole.

*“I think the student clinic could be amazing at all of this [reflective practice]... there’s the evidence-based tasks that they do for new patients or presentations that come through, certainly encourage it.” (Interviewee 5).*

*“…whilst that is they look at it very much as a chore, it still has them thinking outside the box and reflecting on why they have selected the treatment techniques and how that relates to the different best practice evidence. And as a lifelong learner, I think they see very quickly their shortfalls when it comes to clinical practice.” (Interviewee 2).*

Clinical educators reported that student communication with educators is one of the strongest skills students present with. However, an aspect that can be improved is the communication skills of the clinical educators and how they can enhance their communication to assist in the development of the students. Educators are provided with a single training day to enhance their teaching skills. However, the training days are not utilised to their full potential by all educators.

*“Even just a two hour short course, one hour short course on something like that [on communication] would be extremely effective in developing our own communications skills still.” (Interviewee 3).*

*“It’s kind of that one of like [sic], even I suppose that training day, we’re talking about, like, if clinicians aren’t going to them, then you know, they don’t know what to do.” (Interviewee 1).*

A topic that surfaced in a number of interviews was the lack of consistency between clinical educators. This was mentioned as a key contributing factor to the limited use of clinical guidelines when developing an evidence-based management plan for the treatment of patients. The interviewees reported that the expectations the clinicians hold the students to needs to be clear and consistent.

*“Starting with clinicians, and then starting with processes and guidelines that should be introduced into the student clinic to stay consistent across the board. Consistency is key.” (Interviewee 3).*

*“Following OBA [Osteopathy Board of Australia, registration body] guidelines is influenced by clinicians, but the clinic is a good place to become familiar with the guidelines.” (Interviewee 6).*

Participants acknowledged that they, along with the students, need to improve both in terms of professionalism and ‘buying into’ the function of the clinic to enhance the quality of education that the students experience. Multiple interviewees pointed to themselves and their role as a clinical educator as a starting point for improvements in these areas. Whether it be that they need to drive the students to improve their professionalism and commitment, or if the educators themselves need more buy-in and professionalism to be able to perform at higher levels were described.

*“…a student clinic needs to be more professional than what you would see in a normal osteopathic clinic in private practice, just because the public sees you, they know that it’s a student clinic. So you want it to be more professional, so you’re really putting them [patients] at ease. And so from a professionalism point of view, it definitely does develop their skills like they learn, working the [reception] desk, they learn, you know, how to take appointments, how to speak professionally on the phone, how to do record keeping, and how to, the privacy information” (Interviewee 2).*

*“The clinicians need to be able to drive that [professionalism] and the clinicians need to be aware that this is what we’re aiming for... I don’t think that’s there at the moment.” (Interviewee 6).*

### Challenges to and of the SLC environment

The SLC is a suitable learning environment for students to develop integrated healthcare skills across the clinical experience from first patient contact (eg. managing bookings) to resolution of their condition (eg. patient treatment and care) and/or referral to other providers. However, the SLC is a difficult setting to develop relationships with different healthcare professionals due to time pressures, a lack of drive from clinical educators, and a lack of patient diversity.

*I think the students that go to the off campus, you know, the, the community, the community clinics, that they get a better exposure, or more likely get a better exposure because there are other professionals around and often those patients are chronic…. (Interviewee 8).*

A frequently discussed challenge of the SLC environment was the number of patients attending the clinic. The clinical educators reported that it had been an ongoing issue affecting the experience levels of the students and resulted in gaps in the clinical reasoning of students. It was suggested that developing a plan to boost patient numbers would be beneficial to student development.

*“They are not always exposed to a huge amount of patients in the student clinic.” (Interviewee 9).*

The lack of cultural diversity, and clinical presentations, within the limited population of the SLC is another aspect of the environment perceived to impact on student development. Students are often treating a reasonably healthy population, which frequently includes other young osteopathy students. Clinical educators suggested that this resulted in the students becoming efficient at managing mostly routine musculoskeletal presentations. The educators believe that this does not prepare the students for managing patients with more complex care needs that the student will likely encounter in professional practice.

*“I do think we’re quite good at being able to manage uncomplicated patients... when it comes to the yellow flag element, patients that have comorbidities with mental illness, and also those that have got complex neurological conditions and things like that, where you’d have a different degree of expertise in terms of your management….” (Interviewee 9).*

Alternatively, the community-based clinics used within the osteopathy course are thought to be advantageous for gaining experience in treating a variety of patients with comorbidities or impaired cognitive function. This leads to improved communication skills, management of patients with more complex care needs and greater consideration about the indications and contraindications to manual therapy techniques.

*“I think in terms of tutorials, and also the community clinics, there, you do get a decent amount of exposure.” (Interviewee 9).*

The electronic health records system used in the clinic was another challenge for the students recognised by the clinical educators. The system was described as very structured and thorough but this resulted in the students’ reading from a script, rather than engaging with the patient and their individual complaint. This, in turn, impacts the student’s time efficiency.

*“It’s probably not the most user friendly for them. I know that they become quite articulate with it and get to know quite well, but maybe that maybe [sic] that’s a barrier to completing it [note taking] in a timely manner.” (Interviewee 6).*

*“I mean, the software they use at [institution] is awkward, and cumbersome, and you have to know how to work it, which in some ways is annoying and dumb, but I guess it forces you to learn how to adapt to situations that are ideal, and that’s always going to be the case” (Interviewee 8).*

Leadership within the environment is another identified aspect that is often challenging. Many of the participants believe that leadership is dependent on the student personality and is automatic or natural for some. Although not always present in the clinic, the clinical educators believe the clinic does provide the opportunity to display and naturally develop leadership qualities.

*“I think there’s an opportunity for students to lead by example, and lead their peers in terms of helping them with their clinical skills, there’s definitely an opportunity to do that, and there’s probably probably [sic] hasn’t been utilised enough.” (Interviewee 6).*

## Discussion

Our work highlights aspects of the SLC that osteopathy clinical educators perceive the most important in developing students to ensure that they meet the expected standards for osteopathic practice in Australia [33]. This study is the first to explore the perceptions of clinical educators on whether SLCs provide an environment to develop professional capabilities in pre-professional osteopathy students. These capabilities outline the attributes, knowledge and skills that Australian osteopaths are expected to demonstrate in order to practice safely and ethically [33].

Education within a clinical setting provides students with opportunities to develop their skills, attitudes, values and knowledge, and to prepare them for clinical practice [34]. Clinical educators in our study expressed that the osteopathy SLC is a suitable environment to develop and consolidate several capabilities required for osteopathic practice. Consistent with others [35, 36], capabilities that were commonly developed in this environment included the development and implementation of treatment and management plans, enhanced clinical reasoning, and the students’ ability to utilise effective non-verbal and verbal communication strategies.

Students in our course learn to develop and implement osteopathy management plans through classroom learning and simulation [37]. However, it is not until they enter the SLC in a patient care role that they have an opportunity to develop and implement plans in ‘real life’ [38]. Development and implementation of effective management plans, and clinical reasoning, in this context may be associated with the ‘safety in learning’ concept articulated by Bostick, Hall and Miciak [39] – the SLC as a safe, supported environment for experiential learning. Likewise, student communication capability was reported to improve over the time students spent in the SLC. This observation with respect to improved communication through participation in the SLC environment is consistent with others [40–42]. These key elements of health professional practice – implementing treatment and management, clinical reasoning and communication - appear to be supported, and developed by, the SLC environment.

Clinical educators identified the need for them to foster a learning environment in the SLC that optimises the students’ preparedness for professional practice. Key to this was consistency between clinical educators in relaying to students the professional behaviours and capabilities expected in a clinical environment – being a reflective, evidence informed, communicative and professional practitioner. Clinical educators in the work by Levy, Sexton, Willeford, Barnum, Guyer, Gardner and Fincher [43] identified effective communication, constructive feedback, creating a student-centred environment and clinical educator training all assist in developing students adequately for private practice. The vast majority of students in osteopathy programs in Australia will enter private practice upon graduation [20], so ensuring they are prepared for this environment is paramount.

Capacity to engage in reflective practice is a key attribute for successful health professions practice [44]. The SLC environment allows students to develop, and engage with, reflective practice, particularly where there is an opportunity to do so with peers and the clinical educator [45, 46]. Consistent with Albinsson, Elmqvist and Hörberg [45], clinical educators in our study identified that the SLC afforded an opportunity to reflect, however there was recognition of their role in ensuring that students have an opportunity to reflect on the breadth of their practice. An additional opportunity identified by the clinical educators was engagement in EBP. Transfer of EBP skills from classroom to clinical practice is challenging but can be assisted through deliberate task design [47]. Students in our SLC undertake specific EBP tasks associated with patient care. However, the value and effectiveness of these tasks in changing current and future practice has yet to be investigated in this environment, albeit it is a capability for practice [33].

Providing students with opportunities to experience a diverse range of case presentations is a challenge to the SLC environment at our institution. Before commencing their clinical placements, students are taught and assessed on the clinical reasoning and management of a range of common musculoskeletal presentations. More complex presentations, including those with significant psychosocial influences, are taught through simulation [37, 48]. Clinical educators perceived the population seeking care in the SLC to be comprised of younger patients with relatively straightforward clinical presentations. This observation appears to be relatively consistent with audit data from the SLC [29]. To provide students with experience in managing more diverse presentations, clinical educators suggested that students should be required to partake in community clinic placements to support their preparation to deal with the unusual patient they are likely to encounter in professional practice. How these community placements contribute to increased exposure to a range of more complex patient presentations warrants additional research.

Broadly, professionalism and professional practice was consistently identified by the clinical educators as one aspect that requires improvement. The clinical educators, in some instances, identified their role in developing professionalism through role modelling – although not explicitly described as such. Role modelling is widely accepted to be a key teaching and learning strategy for clinical educators [49, 50] and appears to be so in our SLC [51]. The clinical educators also appeared to reflect on how their own performance, and that of their peers, impacted on learning in the SLC. Specifically, the clinical educators identified explicit professional development in their own communication and supervisory skills. It appears that few clinical educators in our SLC have undertaken formal professional development [52] so there is an opportunity to provide informal professional development activities as a starting point. A proactive approach to upskilling clinical educators in skills specific to their educational role provides an opportunity to optimise the SLC environment.

Although the SLC environment appeared to contribute to developing the ‘osteopath’ and ‘communicator’ roles in the Capabilities [33], other roles were not developed to the same extent. Clinical educators appeared to use differing conceptions of leadership in our study whether it be related to working with peers or near-peers or demonstrating leadership attributes in patient care. However, there was an appreciation that the SLC could be used to develop the ‘leadership’ role and further research could be directed towards how best to achieve this. The ‘educator and health promoter’ role was not developed to the same extent as other roles. The clinical educators identified that students were adept at educating patients about exercise as part of their management. Exercise combined with manual therapy is an effective, evidence-based strategy to manage musculoskeletal complaints [53]. However, the broader health promotion role was not considered by the clinical educators. This observation suggests additional work should be directed towards understanding curricula design on how best to develop the health promotor role in pre-professional osteopathy students. The ‘collaborative practitioner’ role also appeared to be one that was challenging to develop in our SLC. Clinical educators suggested that exposure to other health professionals and interprofessional (IP) care was particularly limited and there are no other health professions training in the same SLC as the osteopathy students in our institution. The SLC environment is widely used to develop student IP care capabilities [54] so this appears to be a significant gap. Addressing this issue may require alternative placement opportunities (including private practices and community-based clinics) or institution-wide changes to foster IP education and practice.

The limited cohort of educators in this study, indicates future research could explore the insight of educators from other institutions, as well as canvassing the perceptions of employers, given that the majority of Australian osteopathy students will be employed in private practice upon graduation. Further, research could include exploration of the influence of professional development of clinical educators in the SLC context, and the broader osteopathy clinical learning environment to understand the barriers and enablers to high-quality clinical education and preparation for practice. The authors did not return transcripts for verification or seek feedback from the participants given saturation of the data and the unlikely scenario individual responses would change the outcomes presented here.

## Conclusion

The perception of clinical educators is that the student-led osteopathy clinical learning environment appears to play a positive role in preparing students for practice, particularly the “osteopath” and “communicator” roles in the Capabilities for Osteopathic Practice. The SLC environment also engages students in evidence-based practice and care, as well as providing an opportunity for them to engage in reflective practice. However, the lack of interprofessional learning and care opportunities, and engagement with health promotion activities, is a significant limitation of the current structure of the SLC. These elements of the SLC require addressing to ensure that students are prepared for their role as a health professional.

## Data Availability

The transcripts supporting the conclusions of this article are available on reasonable request from the corresponding author.

## Abbreviations

EBP: Evidence-based practice
SLC: Student-led clinic
VU: Victoria University
